# Leukocyte telomere dynamics in the elderly

**DOI:** 10.1007/s10654-013-9780-4

**Published:** 2013-02-21

**Authors:** Troels Steenstrup, Jacob v. B. Hjelmborg, Laust H. Mortensen, Masayuki Kimura, Kaare Christensen, Abraham Aviv

**Affiliations:** 1 Department of Biostatistics, Institute of Public Health, University of Southern Denmark, Odense, Denmark; 2 Section of Social Medicine, University of Copenhagen, Copenhagen, Denmark; 3 The Center of Human Development and Aging, New Jersey Medical School, University of Medicine and Dentistry of New Jersey, Room F-464 MSB, 185 South Orange Ave, Newark, NJ 07021 USA; 4 The Danish Aging Research Center, University of Southern Denmark, Odense, Denmark; 5 Department of Clinical Biochemistry and Pharmacology, Odense University Hospital, Odense, Denmark; 6 Department of Clinical Genetics, Odense University Hospital, Odense, Denmark

**Keywords:** Telomeres, Aging, Sex, Longitudinal, Cross-sectional

## Abstract

Limited data suggest that leukocytes of the elderly display ultra-short telomeres. It was reported that in some elderly persons leukocyte telomere length (LTL) shows age-dependent elongation. Using cross-sectional and longitudinal models, we characterized LTL dynamics in participants of the Longitudinal Study of Aging Danish Twins. We measured LTL by Southern blots of the terminal restriction fragment length (TRFL) in 476 individuals (73–94 years) in a cross-sectional evaluation and in a subset of this cohort comprising 80 individuals (73–81 years at baseline) who were followed–up for approximately 10 years. Based on the mean TRFL, we found that a) the average rate of LTL attrition was respectively, 27 bp/year (*P* < 0.001) and 31 bp/year (*P* < 0.001) for the cross-sectional and longitudinal evaluations, and b) mean TRFL was 180 bp (95 % CI 43, 320) longer in females than males (*P* < 0.010). For the TRFL distribution, which captures telomeres of all lengths in the DNA sample, we observed significant shifts with age toward shorter telomeres. Based on the measurement error of the TRFLs, we computed that in the longitudinal evaluation 10.6 % of individuals would manifest LTL elongation over 10 years, assuming a 340 bp attrition during this period. This was not significantly different from the empirical observation of 7.5 % of individuals showing LTL elongation. We conclude that accumulation of ultra-short telomeres in leukocytes of the elderly reflects a shift toward shorter telomeres in the entire telomere distribution. Measurement error is the probable explanation for LTL elongation in longitudinal studies.

**Electronic supplementary material:**

The online version of this article (doi:10.1007/s10654-013-9780-4) contains supplementary material, which is available to authorized users.

## Introduction

At any age, leukocyte telomere length (LTL) reflects the highly variable LTL among newborns [1, 2] and its age-dependent shortening after birth. Cross-sectional evaluation of LTL in individuals of different ages provides the overall rate of LTL attrition in the cohort [3–9]. To examine the inter-individual variation in age-dependent LTL attrition, recent studies have used a longitudinal design based on baseline and follow-up LTL measurements in the same individuals [10–20]. The sample size of these studies ranged from 14 [16] to 959 [14]; participants’ age (at baseline) ranged from 5 years [18] to 89.8 years [16]; and the follow-up period lasted from 6 months [19] to 12.4 years [17]. Of these only one relatively small study focused on the elderly [16].

Based on cross-sectional analysis of Southern blots of the TRFLs and single telomere length analysis, persons aged 90–104 years, who are categorized as the oldest old, seem to accumulate ultra-short (<3 kb) telomeres in their leukocytes [21]. However, a longitudinal evaluation of telomere length attrition in peripheral blood mononuclear cells (PBMCs) in the oldest old also concluded that a subset of these subjects displayed telomere elongation [16], a finding in line with results reported in longitudinal studies with younger subjects [10–15, 17–19]. Whether using a quantitative PCR (qPCR)-based method to measure leukocyte telomere DNA content [10, 11, 14–16, 18, 19] or Southern blot analysis [12, 13, 17, 20], these studies reported the age-dependent change in only the mean length of leukocyte telomeres. But the overall distribution of LTL in each DNA sample [21, 22] might provide additional information about LTL dynamics.

In the present study, using Southern blots of the TRFLs and both cross-sectional and longitudinal designs we explored the following questions: First, do elderly persons display an accumulation of ultra-short telomeres in their leukocytes? Second, is there sufficient evidence to support the concept that a subset of elderly persons does not display age-dependent LTL shortening?

## Methods

### Subjects

Our subjects come from the Longitudinal Study of Aging Danish Twins (LSADT). For the cross-sectional evaluation, we studied 476 same-sex twins (238 twin pairs), 152 males (73–93 years), 324 females (73–94 years). The twins were stratified into four groups, based on sex and age (upper and lower halves of age distribution for males and females) (Table 1). The mean age difference between the older and younger male groups was 5.7 years, whereas the mean age difference between the older and younger female groups was 7.1 years.

**Table 1:** Age characteristics of the four cross-sectional groups

Groups	Number	Mean age	SD
Females			
50 % younger	162	76.1	1.4
50 % older	162	83.2^a^	3.8
Males			
50 % younger	76	75.8	1.0
50 % older	76	81.5	3.1

^a^Indicates a statistically significant difference between males and females

Of the 476 twins, 80 twins had baseline and follow-up TRFL measurements (Table 2). The mean age difference between baseline and follow-up was 10.9 years (95 % prediction interval: 10.1, 11.6). Of these twins, 24 were males (73–79 years at baseline) and 56 were females (73–81 years at baseline).

**Table 2:** Age characteristics of the longitudinal survey

Groups	Number	Mean age	SD
Females			
Baseline	56	75.1	1.8
Follow-up	56	85.9	1.8
Males			
Baseline	24	75.6	1.7
Follow-up	24	86.5	1.7

This research was approved by the scientific-ethical committee for Vejle and Funen counties, and all participants provided written informed consent.

### Leukocyte TRFL measurements

Telomere length was measured by Southern blots of the TRFLs as previously described [23]. This approach yields the TRFL distribution for each DNA sample. Unless indicated, each sample was measured in duplicate (performed on different gels). For the cross-sectional evaluation, samples from the co-twins in each twin pair were resolved in adjacent lanes of the same gel, while for the longitudinal evaluation, samples from the co-twins were randomized, but baseline and follow-up samples for each co-twin were resolved in adjacent lanes. The inter-assay coefficient of variation for the 476 twins was 3.1 % (95 % CI 2.8, 3.3 %) and 2.8 % (95 % CI 2.5, 3.2 %) for the 80 twins. These values differ from those in a previous publication based on the same individuals [22]. This is explained by a difference in the way the DNA samples were digested. For this work, the DNA was digested using the *Hinf*I/*Rsa*I enzyme, whereas *Hph*I/*Mnl*I was used in the previous work [23].

From a set of TRFL distributions we obtained the mean distribution by calculating the mean height of the distribution for each value of the molecular weight (MW). This approach was undertaken for four subsets of the participants in the cross-sectional analysis (upper and lower halves of age distribution for males and females) and in the longitudinal analysis (baseline and follow-up for males and females).

### Statistical analysis

For cross-sectional mTRFL measurements we have used the following random effects model:

1 $$ {\text{mTRFL}}_{\text{ikl}} = \, \beta + \beta_{\text{sex}} *{\text{sex}} + \beta_{\text{age}} *{\text{age}} + \eta_{\text{l}} + \eta_{\text{kl}} + \varepsilon_{\text{ikl}} $$

which we fitted to the measurements of the 476 individuals. Index *l* identifies the twin pair, index *k* identifies the specific twin within the pair and index *i* identifies the specific repeated measurement (most DNA samples were measured twice). The variable “age” refers to the age of the individual at the time of the DNA sample collection and “sex” is 0 for males and 1 for females.

For longitudinal mTRFL measurements we have used the following random effects model:

2 $$ {\text{mTRFL}}_{\text{ijkl}} = \beta + \beta_{\text{sex}} *{\text{sex}} + \beta_{\text{age}} *{\text{age}} + \beta_{\text{occ}} *{\text{occ}} + \eta_{\text{l}} + \eta_{1}^{\prime } *{\text{occ}} + \eta_{\text{kl}} + \eta_{\text{kl}}^{\prime } *{\text{occ}} + \varepsilon_{\text{ijkl}} $$

which we fitted to the repeated measurements of the 80 individuals undergoing the longitudinal analysis. For this analysis, index *l*,*k* and *i* have the same meaning as in the cross-sectional analysis, but index *j* identifies whether the DNA was from the baseline or follow-up sample. The variable “occ” is binary and has value 0 if the DNA was from the baseline sample and the value 1 if the DNA was from follow-up sample. The variable termed “age” refers to the age of the individual at the time of the baseline sample collection.

Some variation between samples might relate to the resolving of DNA samples on different gels. The term “gel-effect” is used to describe this phenomenon, which is assumed to affect all mTRFL measurements on a single gel by the same amount (although this amount might differ from one gel to the other).

We estimated the within-sample (or inter-assay) coefficient of variation as the ratio of the square root of the residual variation to the overall mean, where we find the residual variation using the above mentioned random effects models for the cross-sectional and longitudinal evaluations, respectively. A direct method for estimating the residual variation *s*, is to take the weighted sum of the individual variances divided by their weights, i.e.,

3 $$ {\text{s}}^{2} = \, \left[ {\Upsigma_{i} \left( {{\text{n}}_{\text{i}} - \, 1} \right){\text{s}}_{\text{i}}^{2} } \right]/\left[ { \, \Upsigma_{\text{i}} \left( {{\text{n}}_{\text{i}} - \, 1} \right)} \right] $$

where *n*_*i*_ is the number of repeated measurements for individual *i* and *s*_*i*_ is this individual’s estimated standard deviation based on those *n*_*i*_ measurements. These two methods produce nearly identical estimates of the coefficient of variation (there was no difference up to three significant digits).

From sets of individual TRFL distributions we obtained mean distributions, which we then compared by examining differences between the two data sets. We did this separately for males and females with cross-sectional data, where we compared the 50 % younger with the 50 % older individuals, and with longitudinal data, where we compared baseline with follow-up measurements. We used a nonparametric permutation test to compare the difference between mean distributions from two certain groups to that of two groups whose members were randomly assigned. The boundaries of the TRFL distribution were fixed at 2 kb for the lowest MW, i.e., the shortest TRFL and 30 kb for the highest MW, i.e., the longest TRFL.

Unless otherwise specified, statistically significant denotes *P* < 0.05. All statistical analyses were performed using either Stata 11 (StataCorp LP, College Station, Texas, USA) or R (The R Project for Statistical Computing).

## Results

### Cross-sectional and longitudinal evaluation of the mean TRFL

We derived from the TRFL distributions the following parameters: mTRFL, the mean values of the lower 50 % TRFL and 25 % TRFL (mTRFL_50_ and mTRFL_25_, respectively) and the mode (MTRFL) for the 476 twins in the cross–sectional evaluation (Table 3) and those of the subsample consisting of 80 twins in the longitudinal evaluation (Table 4). Here we present only results of our analysis of the mTRFL, a value that corresponds to LTL, because the analysis of the other derived parameters of the TRFL distributions yielded no additional information over and above that based on the mTRFL analysis.

**Table 3:** Sample summaries of the cross-sectional evaluation

Parameter	1997	95 % CI
Females^a^		
MTRFL	4.5	4.4–4.6
mTRFL	5.8^c^	5.7–5.8
mTRFL_50_	3.9	3.9–4.0
mTRFL_25_	3.2	3.2–3.3
Males^b^		
MTRFL	4.4	4.3–4.5
mTRFL	5.6	5.5–5.7
MTRFL_50_	3.8	3.8–3.9
mTRFL_25_	3.2	3.2–3.3

TRFL values are in kb. Parameters of the TRFL distribution include: the mode (MTRFL), the mean (mTRFL), the mean values of the lower 50 % TRFL (mTRFL_50_) and the mean of the lower 25 % TRFL (mTRFL_25_)

^a^324 individuals (560 observations—two measurements for most individuals) with age range 73–94

^b^152 individuals (248 observations—two measurements for most individuals) with age range 73-93

^c^Indicates a statistically significant difference between males and females

**Table 4:** Sample summaries of the longitudinal evaluation

Parameter	1997	2007	Diff.	P value	95 % CI
Females^a^					
MTRFL	4.7	4.4	−0.24	<0.001	−0.31 to −0.18
mTRFL	5.9	5.6	−0.37	<0.001	−0.42 to −0.30
mTRFL_50_	4.0	3.8	−0.19	<0.001	−0.24 to −0.15
mTRFL_25_	3.3	3.2	−0.13	<0.001	−0.17 to −0.088
Males^b^					
MTRFL	4.5	4.3	−0.15	0.004	−0.25 to −0.048
mTRFL	5.6	5.4	−0.26	<0.001	−0.37 to −0.16
mTRFL_50_	3.8	3.7	−0.12	0.001	−0.19 to −0.044
mTRFL_25_	3.2	3.1	−0.059	0.052	−0.12 to −0.000

TRFL values are in kb. Parameters of the TRFL distribution include: the mode (MTRFL), the mean (mTRFL). the mean values of the lower 50 % TRFL (mTRFL_50_) and the mean of the lower 25 % TRFL (mTRFL_25_)

^a^56individuals (112 observations—two measurements per individual) with baseline age range 73–81 24

^b^24 individuals (48 observations—two measurements per individual) with baseline age range 73-79

**Fig. 1:**
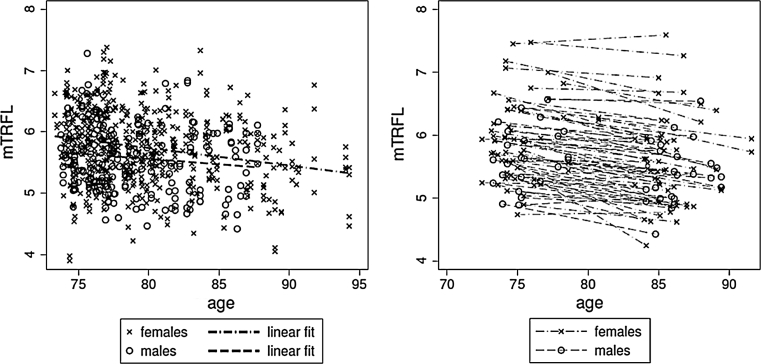
Scatter plot of mTRFL versus age. Cross-sectional data are shown in the *left panel* and longitudinal data are shown in the *right panel*. In the *left panel*, the regression lines are for females (*steeper slope*) and males. In the *right panel*, the lines connect baseline and follow-up measurements for each individual

Scatter plot of mTRFL from the 476 individuals as a function of age for males and females showed considerable inter-individual variation (Fig. 1, left panel). Age adjusted mTRFL was 180 (95 % CI 43, 320) bp longer in females than males (*P* < 0.010). The overall rate of age-dependent mTRFL shortening was 27 bp/year (95 % CI 12, 42). The rate of age-dependent mTRFL shortening was 29 bp/year (95 % CI 12, 47) for females and 18 bp/year (95 % −12, 49) for males with no evidence for a sex-related difference in mTRFL shortening (*P* < 0.529).

The longitudinal data from the repeated measurements (approximately 10 years apart) of the 80 individuals also showed considerable inter-individual variation (Fig. 1, right panel). To illustrate the individual change in mTRFL, baseline and follow-up mTRFLs are linked with a line. The overall rate of age-dependent mTRFL shortening was 31 bp/year (95 % CI 26, 37). The rate of age-dependent mTRFL shortening was 34 bp/year (95 % CI 28, 41) for females and 24 bp/year (95 %: 14, 34) for males, with no evidence for a sex-related difference in mTRFL shortening (*P* < 0.082). There was no effect of baseline age on mTRFL shortening (*P* < 0.741). 

**Fig. 2:**
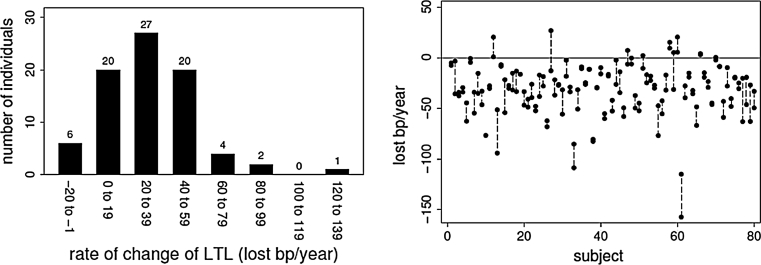
Rate of change in the mTRFL. Histogram of the distribution of the rate of change in mTRFL for 80 individuals is displayed in the *left panel*. Rate of change based on each of duplicates of baseline and follow-up samples from 78 individuals and one measurement in two individuals are shown in the *right panel*. The duplicates of measurements of a given sample are presented as two data points connected by *vertical lines*. The 6 Individuals displaying mTRFL lengthening are those with a negative value of lost bp/year (1st *bar* in the *left panel*), or equivalently, those for which the mean value of the two repeated measurements lie above the *horizontal line*, which represents 0 change in mTRFL (*right panel*)

Six individuals (7.5 %) displayed a longer mTRFL in follow-up than in baseline samples (Figs. 1, right panel, 2, left panel). Among these 6 individuals, only in 4 both duplicate measurements of mTRFL showed lengthening (Fig. 2, right panel). (Notably, because of insufficient DNA, there were no replicate measurements of the TRFL distribution for 2 individuals.) Based on the estimates of the components in the random effects model, we computed that 10.6 % of individuals should manifest mTRFL lengthening over a 10 year period, which is not a significant deviation from the empirical finding of 7.5 % (*P* < 0.468).

### Cross-sectional and longitudinal evaluation of TRFL distributions

**Fig. 3:**
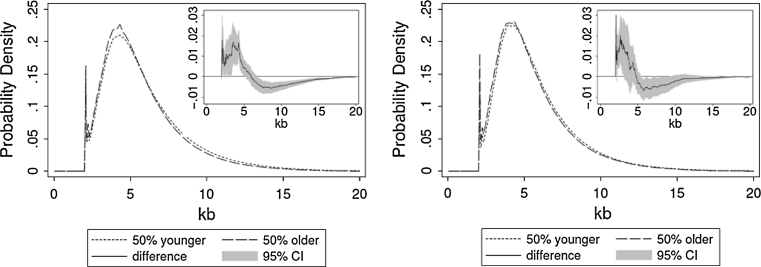
Average TRFL density functions for the older versus the younger participants based on the cross-sectional analysis. Females are shown in the *left panel* and males in the *right panel*. The differences between TRFL density functions (*shaded area*- 95 % CI) are shown in the *insets*. Older persons show an under-representation of longer telomeres and an over-representation of shorter telomeres than younger ones. This is evident by the difference (95 % CI) between the distribution curves of samples from older versus younger participants

The cross-sectional data of the TRFL distribution showed that the older subsets of females and males (upper halves of age distribution) displayed an under-representation of longer telomeres and an over-representation of shorter telomeres than younger subsets (lower halves of age distribution), more so in the female groups than in the male groups (Fig. 3). These findings were expected, since the age difference was smaller among the male groups. Although the shift of a given TRFL towards a lower MW with age seemed small, the cumulative effect of the overall shift throughout the TRFL distribution was considerable. This phenomenon was clearly demonstrated by the difference (95 % CI) between the distribution curves of older versus younger participants. A nonparametric permutation test indicated that the difference of the TRFL distributions between the upper and lower halves of the age distributions were unlikely to be due to chance alone (for males, randomly generated groups displayed a difference larger than that displayed by the two original groups in less than 20 out of 1,000 times; for females it was less than 1 in a 1,000).

**Fig. 4:**
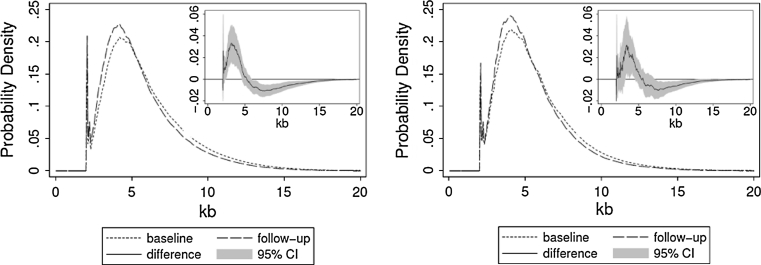
Average TRFL density functions for baseline versus follow-up analyses. Females are shown in the *left panel* and males in the *right panel*. The differences between TRFL density functions (*shaded area*- 95 % CI) are shown in the insets. Follow-up TRFLs show an under-representation of longer telomeres and an over-representation of shorter telomeres than baseline TRFLs. This is evident by the difference (95 % CI) between the TRFL distribution curves between baseline and follow-up samples

For the 80 individuals participating in the longitudinal evaluation, after 10 years there was a shift towards increased representation of shorter telomeres and diminished representation of longer telomeres in the TRFL distributions (Fig. 4). Again, a nonparametric permutation test showed that the difference between baseline and follow-up exams was unlikely to be due to chance alone (for males, randomly generated groups displayed a difference larger than that displayed by the two original groups in less than 50 out of 1,000; for females it was less than 1 out of 1,000).

## Discussion

The central finding of this work is that in the elderly age-dependent LTL shortening is essentially the same as that in younger individuals, based on published studies [3–6]. The accumulation of ultra-short telomeres in the very old [21] largely reflects a shift towards shorter telomeres throughout the entire distribution of telomere lengths in each given DNA sample. This conclusion is based on both the mTRFL and the overall distribution of the TRFLs, whether derived from the cross-sectional or the longitudinal evaluations.

Notably, at baseline, the participants in this study were mostly younger than 90 years; as such they are not categorized as the oldest old. It is doubtful that finding in this population does not apply to older individuals. In addition, the participants in the longitudinal arm of this study survived for an average of 10 years. Thus, they are a selected group. However, the conclusions derived from the cross-sectional evaluations and the longitudinal ones are the same with regard to the age-dependent attrition in mTRFL, i.e., LTL, and the entire TRF distribution. Moreover, in this study the lowest scanning limit of the TRF distribution was fixed at 2 kb, although on some occasions the TRF signal (above background) clearly extended to below 2 kb. Thus, our conclusions are confounded by the inability to capture TRFs below the 2 kb limit in older versus younger participants in the cross-sectional evaluation and in the follow-up versus baseline examinations in participants in the longitudinal evaluation. That said, the TRFs consist of both the canonical (strictly TTAGGG repeats) region and a non-canonical region up to the nearest restriction sites (the so-called X-region). Given that the X-region has been recently estimated to be between 1 and 2 kb, depending on the restriction enzyme used to generate the TRFs [24], it is safe to conclude that the TRF distribution captures most of the ultra-short telomeres in the DNA samples. Thus, our findings clearly show that as individuals age, their overall LTL is determined by less contribution of longer telomeres and more contribution of shorter telomeres, including ultra-short telomeres, across most if not the entire range of the canonical part of the TRFL distribution.

As per previous studies in younger subjects [3, 5, 6, 20], females had a longer age-adjusted LTL than males, but we did not find evidence for a slower age-dependent LTL shortening among females than among males. Several studies observed that the rate of LTL shortening is proportional to baseline LTL, such that individuals with a longer LTL at baseline have a faster rate of age-dependent LTL shortening [13–15]. However, concerns have been raised that these findings are at least in part a consequence of mathematical coupling [25].

Both theoretical considerations and empirical data point to measurement error of telomere length as the main culprit for what seems to be LTL elongation in a small subset of participants in the longitudinal evaluation of age-dependent LTL shortening. This conclusion makes intuitive sense, given that LTL dynamics mirror telomere dynamics in hematopoietic stem cells (HSCs) [26] and as telomerase activity is too small or absent altogether in HSCs during extra-uterine life to prevent age-dependent LTL shortening [27–29]. Moreover, although telomerase activity in activated T and B lymphocytes [30] might attenuate the rate of telomere shortening in these circulating cells, it is unlikely to cause LTL elongation with age.

Using both cross-sectional and longitudinal designs, our analyses of the entire telomere length distributions of leukocytes reinforce the conclusions of a previous study in a younger cohort [17]. That study concluded that a larger measurement error of LTL and a shorter interval between baseline and follow-up measurements are the main explanations for LTL elongation in most individuals. Although our study has not focused on the oldest old, it is very unlikely that the findings by others [16] of telomere elongation in PBMCs in a large subset of these subjects reflect a true biological phenomenon rather than being the outcome of measurement error. That is because telomere length measurements by any current method are hopelessly crude when it comes to the assessment of the rate of LTL shortening over time spans of few years rather than few decades.

In conclusion, cross-sectional and longitudinal studies of LTL dynamics, based on the mean length of LTL and telomere length distribution, show that the accumulation of ultra-short telomeres in the elderly reflects the overall shift towards shorter telomeres with age. There is little evidence to support the notion that age-dependent LTL attrition stalls or reverses course in elderly humans.

## Electronic supplementary material

Below is the link to the electronic supplementary material.

Supplementary material 1 (PNG 42 kb)
