# Supplementary figures and images for: Leukocyte telomere dynamics in the elderly
Source: Eur J Epidemiol. 2013 Feb 21;28(2):181–7. doi: 10.1007/s10654-013-9780-4 (PMC3604590; doi:10.1007/s10654-013-9780-4)

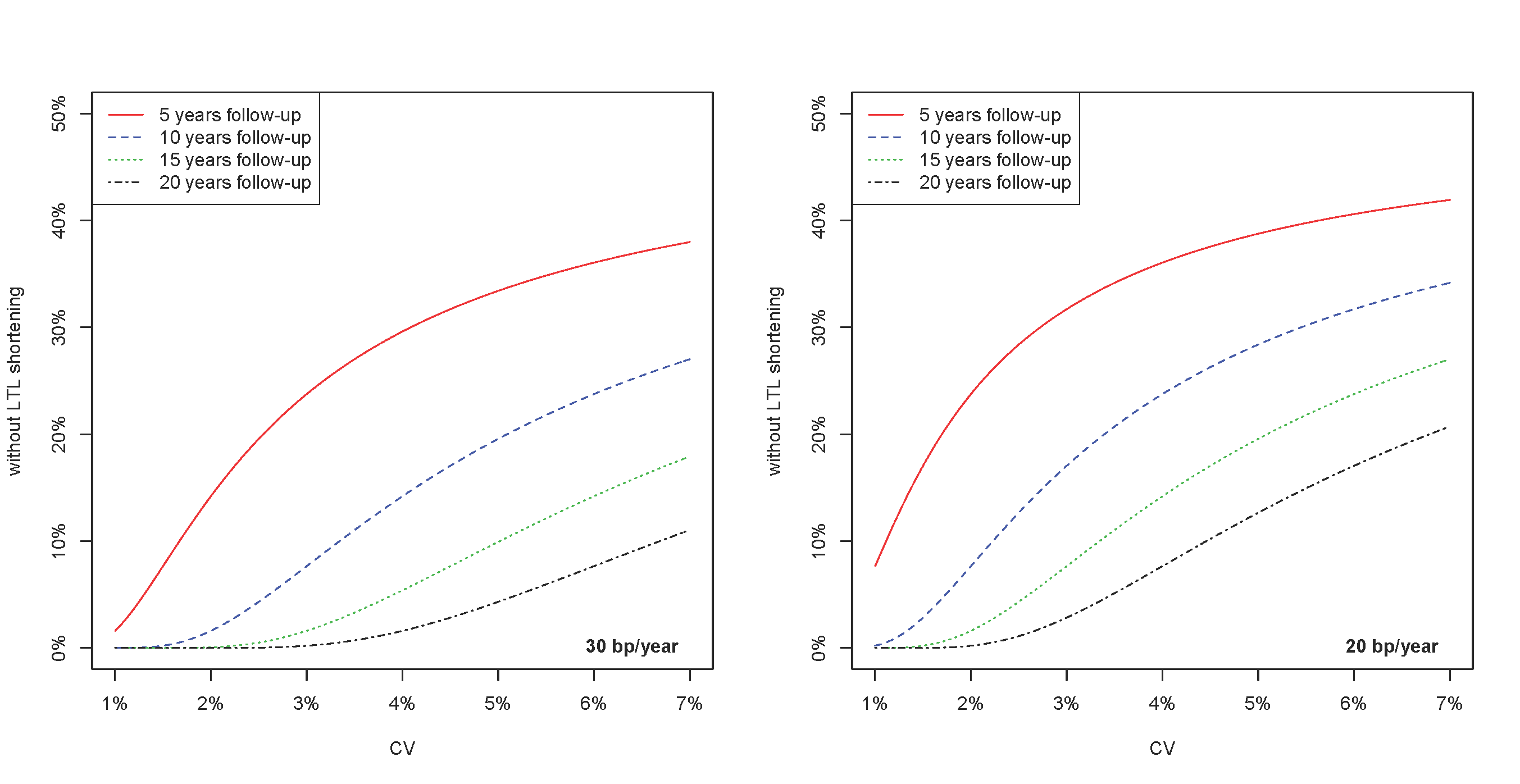

Supplement: Supplementary file 1 — Supplementary material 1 (PNG 42 kb) [file 10654_2013_9780_MOESM1_ESM.png]
